# Application of Machine Learning Algorithms for Prognostic Assessment in Rotator Cuff Pathologies: A Clinical Data-Based Approach

**DOI:** 10.3390/diagnostics13182915

**Published:** 2023-09-11

**Authors:** Umile Giuseppe Longo, Calogero Di Naro, Simona Campisi, Carlo Casciaro, Benedetta Bandini, Ayoosh Pareek, Roberta Bruschetta, Giovanni Pioggia, Antonio Cerasa, Gennaro Tartarisco

**Affiliations:** 1 Orthopaedic and Trauma Surgery, Fondazione Policlinico Universitario Campus Bio-Medico, Via Alvaro del Portillo 200, 00128 Rome, Italy; c.dinaro@unicampus.it (C.D.N.); c.casciaro@unicampus.it (C.C.);; 2 Research Unit of Orthopaedic and Trauma Surgery, Department of Medicine and Surgery, Università Campus Bio-Medico di Roma, Via Alvaro del Portillo 21, 00128 Rome, Italy; 3 Institute for Biomedical Research and Innovation (IRIB), National Research Council of Italy (CNR), 98164 Messina, Italy; simona.campisi@irib.cnr.it (S.C.); roberta.bruschetta@irib.cnr.it (R.B.); giovanni.pioggia@irib.cnr.it (G.P.); antonio.cerasa76@gmail.com (A.C.); 4 Department of Engineering, Università Campus Bio-Medico di Roma, Via Alvaro del Portillo 21, 00128 Rome, Italy; 5 Hospital for Special Surgery, New York, NY 10021, USA; pareeka@hss.edu; 6 S’Anna Institute, 88900 Crotone, Italy; 7 Pharmacotechnology Documentation and Transfer Unit, Preclinical and Translational Pharmacology, Department of Pharmacy, Health Science and Nutrition, University of Calabria, 87036 Arcavacata di Rende, Italy

**Keywords:** rotator cuff, outcome predictors, machine learning, ensemble of classifiers

## Abstract

Aim: The overall aim of this proposal is to ameliorate the care of rotator cuff (RC) tear patients by applying an innovative machine learning approach for outcome prediction after arthroscopic repair. Materials and Methods: We applied state-of-the-art machine learning algorithms to evaluate the best predictors of the outcome, and 100 RC patients were evaluated at baseline (T0), after 1 month (T1), 3 months (T2), 6 months (T3), and 1 year (T4) from surgical intervention. The outcome measure was the Costant–Murley Shoulder Score, whereas age, sex, BMI, the 36-Item Short-Form Survey, the Simple Shoulder Test, the Hospital Anxiety and Depression Scale, the American Shoulder and Elbow Surgeons Score, the Oxford Shoulder Score, and the Shoulder Pain and Disability Index were considered as predictive factors. Support vector machine (SVM), k-nearest neighbors (k-NN), naïve Bayes (NB), and random forest (RF) algorithms were employed. Results: Across all sessions, the classifiers demonstrated suboptimal performance when using both the complete and shrunken sets of features. Specifically, the logistic regression (LR) classifier achieved a mean accuracy of 46.5% ± 6%, while the random forest (RF) classifier achieved 51.25% ± 4%. For the shrunken set of features, LR obtained a mean accuracy of 48.5% ± 6%, and RF achieved 45.5% ± 4.5%. No statistical differences were found when comparing the performance metrics of ML algorithms. Conclusions: This study underlines the importance of extending the application of AI methods to new predictors, such as neuroimaging and kinematic data, in order to better record significant shifts in RC patients’ prognosis. Limitations: The data quality within the cohort could represent a limitation, since certain variables, such as smoking, diabetes, and work injury, are known to have an impact on the outcome.

## 1. Introduction

The most common etiology of shoulder discomfort is rotator cuff (RC) disease, which may account for up to 70% of all consultations involving the shoulder [1,2,3]. It has been demonstrated that RC tears result in substantial discomfort and impairment, as well as reduced performance in daily living tasks [3,4]. Even though surgery is widely accepted around the world [5,6], the possibility of re-tearing is still a significant postoperative obstacle [5,6,7,8,9]. A re-tear is considered a significant risk according to the age, the extent of the first lesion, and the fatty degeneration of the patient [10,11].

About 25% of patients with RCTs experience anxiety or depression, and psychological health may be a key indicator of how well a patient will recover from arthroscopic rotator cuff surgery [12]. Nonetheless, in medical care, the functional assessment frequently focuses on the objective aspects of the illness, such as measuring range of motion (ROM) and strength [12,13]. In light of this, orthopedic research has progressed, and the creation of well-established, patient-oriented metrics has offered clinical outcome evaluation a new perspective, in addition to objective measurements [14]. Patient-Reported Outcome Measures (PROMs) are frequently employed to assess the patients’ wellness [15]. Bypassing the clinician’s evaluation, PROMs are subjective patient-reported scales designed to offer clinical status outcomes [16,17]. Insufficient pain alleviation for patients with shoulder discomfort may lead to the development of mental health issues such as depression and anxiety [18,19]. These psychological disorders represent strong predictors of worse postoperative functional outcomes [20,21]. An example of PROM used as a screening tool to evaluate individuals with musculoskeletal disorders’ psychological health is the Hospital Anxiety and Depression Scale (HADS) [22,23]. The HADS is designed to detect depression and anxiety among non-psychiatric ward patients [24]. Physical symptoms of emotional distress including headaches, weight loss, and insomnia are not included in this questionnaire since they may be the outcome of a medical illness rather than emotional distress itself [25]. The Shoulder Pain and Disability Index (SPADI), on the other hand, was one of the first PROMs to be established specifically for patients with shoulder diseases, to assess pain and impairment [26,27]. Due to its proven test–re-test reliability [28] and change sensitivity, it is commonly used in orthopedic clinical practice [29]. Accordingly, the Simple Shoulder Test (SST) appears to share the same assets [30]. The SST was created to determine the functional limitations of the diseased shoulder in relation to the patient’s day-to-day activities [31]. Again, the 36-Item Short-Form Health Survey (SF-36) is a 36-item questionnaire that is widely included in orthopedics research and represents a valid approach for assessing health-related quality of life [32,33]. It examines the social, emotional, and physical functioning of the patient [34]. The SF-36 is frequently correlated with the Oxford Shoulder Score (OSS) [35]. The latter is a 12-item, subjective questionnaire, based precisely on the parameters of pain and function of the injured shoulder [36]. The OSS consistency, repeatability, and validity have all been established [37]. Finally, the American Shoulder and Elbow Surgeons (ASES) score was designed to evaluate shoulder discomfort and functional limitations in adults with musculoskeletal disorders [38]. The American Academy of Orthopedic Surgeons and the ASES Value Committee have recognized it as an outcome instrument that should be utilized for all shoulder pathology patients due to its applicability [39,40,41].

According to the studies carried out to date, the ASES, DASH, SPADI, OSS, and SST are the most employed questionnaires for the evaluation of the general health of orthopedic patients in both the preoperative and postoperative settings [42]. However, in the current literature, there is no investigation of the reliability of these measures as predictors of outcome in RC patients. The aim of this study was to apply state-of-the-art machine learning algorithms to evaluate the validity of these scales to predict the outcome after arthroscopic repair in RC patients. Longitudinal evaluations at 1, 3, 6, and 12 months from surgical intervention were calculated.

## 2. Materials and Methods

### 2.1. Population

We conducted a prospective cohort study enrolling patients admitted to the outpatient department of Orthopedics from October 2021 to April 2023. Here, 100 patients who underwent arthroscopic rotator cuff repair for rotator cuff tears of any grade were consecutively enrolled. For each patient, demographic information and comorbidities were collected at study entry, as reported in Table 1. Moreover, patients underwent clinical assessment on admission (T0) and after one month (T1), three months (T2), and six months (T3) at discharge, whereas the outcome variable was assessed after twelve months at discharge (T4).

Informed consent was obtained for all the enrolled patients. The study was conducted according to the guidelines of the Declaration of Helsinki and approved by the Institutional Review Board of Campus Bio-Medico University of Rome (COSMO study, protocol number: 78/18 OSS ComEt CBM, 16/10/18). The study was developed following the Good Clinical Practice (GCP) guidelines.

### 2.2. Proposed Approach

As an outcome measure, we used the Costant–Murley Shoulder Score, which is a 100-point scale recommended by the European Society for Shoulder and Elbow Surgery (ESSES) to assess shoulder function. It evaluates four subscales related to shoulder pathology: pain, daily living activities, strength, and range of motion [43]. The higher the score, the better the quality of the function. Then, to make prediction easier for the machine learning algorithms, the outcome variable was divided into four classes (poor, fair, good, and very good), as explained in Table 2.

**Figure 1:**
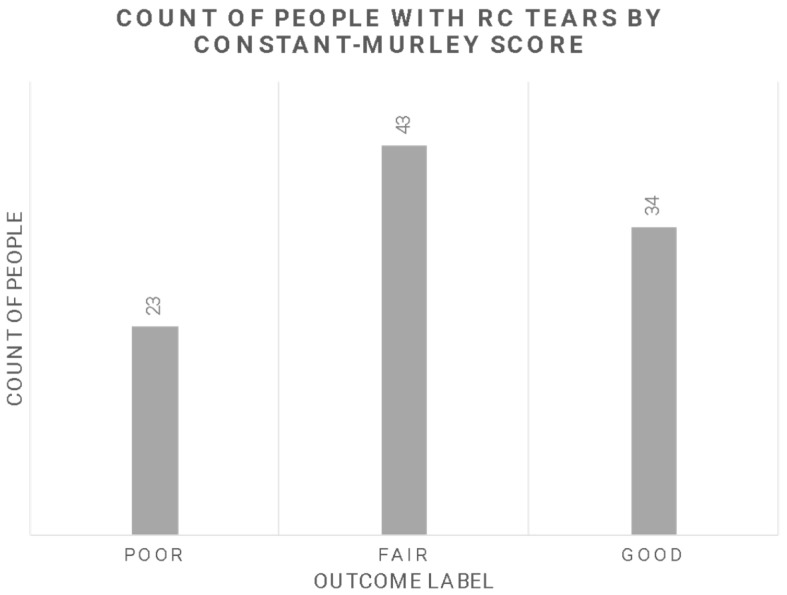
Outcome variable distribution.

By converting the continuous Constant–Murley Score into categorical values, only three classes were obtained, that is poor, fair, and good. No very good class was found (Figure 1).

#### 2.2.1. Predictors’ Selection

As previously mentioned, several clinical scales were tested as predictors of the outcome variable at admission (T0) and during follow-up (T1–T3):

- 36-Item Short-Form Survey (SF-36): A short-form, patient-reported assessment that provides an eight-scale profile of scores for both physical and mental health [44].
- Simple Shoulder Test (SST): A shoulder-specific scale that assesses the affected shoulder’s functional limits in people with shoulder disorders [45]. It includes 12 questions in a dichotomous (yes/no) style, with scores ranging from 0 (worst) to 12 (best).
- Hospital Anxiety and Depression Scale (HADS): A self-reported questionnaire for detecting states of anxiety and depression in a non-psychiatric setting. The anxiety and depression subscales are evaluated using seven questions each, on a four-point (0–3) response scale [46] (Table 3).

- American Shoulder and Elbow Surgeons Score (ASES): A 100-point scale that estimates two dimensions of shoulder function—pain and performance—in activities of daily living. The ASES score allocates 50 points for measuring function and 50 points for pain [47].
- Oxford Shoulder Score (OSS): A 12-item, patient-reported scale created especially for evaluating shoulder surgery results. Each of the 12 questions can be scored from 1 (best) to 5 (worst), so the final score ranges from 12 to 60 [48].
- Shoulder Pain And Disability Index (SPADI): A patient-reported score aimed at quantifying pain and disability in patients with a shoulder injury. It includes 13 items, and there are 2 subscales: pain and disability. The pain subscale has five items, whereas the disability scale consists of eight items. The total score is computed by averaging the 2 subscale scores, thus ranging from 0 to 100 [49].
- Additional demographic and clinical variables, such as age, sex, weight, height, and the Goutallier grade, were also considered potential predictors (Table 1). The Goutallier classification was used to quantify the amount of fatty degeneration of the rotator cuff musculature. It has five stages, starting from Stage 0, meaning normal muscle, to Stage 4, where fat is more present than muscle [50].

The features were chosen based on their proven use in orthopedic patient assessment and their potential to reflect various aspects of patient well-being, pain, and functional ability. We aimed to incorporate a balanced combination of established clinical metrics and additional variables that could contribute to the prediction of the outcome. The aforementioned metrics capture different facets of patient well-being and functional limitations, making them plausible candidates for predicting the outcome.

#### 2.2.2. Statistical Analysis

In this research, we used a ML approach to predict clinical outcomes based on data collected at different times (T0–T3).

Since the variables were not normally distributed, they were compared at admission and during follow-up using the Friedman test, and the Durbin–Conover test for post-hoc pairwise comparison. The Friedman test is suitable for repeated measure data, examining the null hypothesis that there are no differences in the distributions of the variables between the timepoints. The statistical analysis was conducted using the Jamovi statistical software (version 2.3.19.0). We set the significance level (α) at α = 0.05 to determine statistical significance. Table 4 explains the results of the preliminary statistical analysis and the average ± standard deviation of each variable throughout the sessions. The results were highly significant for all variables, with *p*-values consistently lower than 0.001.

#### 2.2.3. Machine Learning Approach

At first, considering the limited number of features provided, all the features were selected as input of various canonical machine learning classifiers, including support vector machine (SVM), k-nearest neighbors (k-NN), naïve Bayes (NB), and random forest (RF), in addiction to logistic regression (LR). All the classifiers were tested separately among each session (T0–T3) to assess their ability to predict the output at one year from discharge.

Additionally, after extensive testing, we discovered that the LR and RF classifiers performed better than the others, offering the best predicting performances as well as additional benefits for our specific objective. In particular, LR is a linear classifier that employs a logistic function to model the relationship between the input features and output classes. Its clarity and interpretability fit the need to comprehend the importance of various factors in making predictions about the course of rotator cuff disease. RF, on the other hand, is an ensemble method that builds a number of decision trees and combines their predictions to reach a decision. The RF classifier was preferred to a simple decision tree (DT) classifier because it mitigates overfitting and captures complex relationships in the data. Furthermore, the ensemble nature of RF further improves its robustness and predictive accuracy, making it suitable for our objective of precise outcome prediction.

Later, correlations between features were computed, keeping only those variables that were highly correlated with the output and poorly correlated with other features. Moreover, a statistical-based feature selection method (Chi-squared test) was performed to both reduce the computational cost of modeling and to assess whether the performances improved with the shrunken set of features. This step aimed at eliminating redundant or irrelevant features, focusing on those with stronger predictive power. All these steps were performed using Python 3 on the Jupyter Notebook platform.

The data preprocessing was firstly performed to treat missing data and scale variables, ensuring the effectiveness of the predictive models. Missing values were replaced with the median value of the predictor. All the variables were then standardized using the RobustScaler module provided in the scikit-learn library, which removes the median value and scales the data according to the quantile range [51], mitigating the effect of outliers.

For each session and for each machine learning model, we employed the nested cross-validation (Nested CV), a procedure for model hyperparameters’ optimization and for preventing overfitting problems. After defining the hyperparameter space, the procedure involves two loops:

- The outer loop uses K-fold cross-validation (K-fold CV) to divide the dataset into training set and test sets, assessing the quality of the model trained on the inner loop.
- The inner loop performs hyperparameter tuning by using K-fold CV on the training set.

The strength of this procedure is that it does not use the same data to model parameters and estimate the performance of the model, reducing the risk of overfitting associated, instead, with traditional cross-validation techniques. The two k values for the inner and outer loops were set to balance the computational cost of the procedure and to provide an unbiased and reliable estimate of the performance. In this direction, we selected a value equal to 10 for the outer loop and 3 for the inner loop [52].

Accuracy was provided as the score of the estimator for each run of the cross-validation. Classification performances were then assessed as mean ± standard deviation across the folds.

For each run of the cross-validation, the estimator’s scores were provided as accuracy, precision, recall, F1-score, and AUC. By calculating recall metrics for each severity category (poor, fair, good), we extended our analysis. This refinement shed light on the model’s proficiency in identifying patients at various severity levels.

Classification performances were then assessed as mean ± standard deviation across the folds (Table 5 and Table 6). This approach provided a thorough understanding of the models’ predictive abilities across various sessions and spotted any potential changes in performance over time.

#### 2.2.4. Feature Selection

A statistical feature selection method was also applied to each session to reduce data dimensionality and explore whether models’ performances improved. Specifically, the ANOVA F-value was computed to evaluate the relationship between each input variable and the output, obtaining a score. The higher the score, the more output-dependent the variable is.

**Figure 2:**
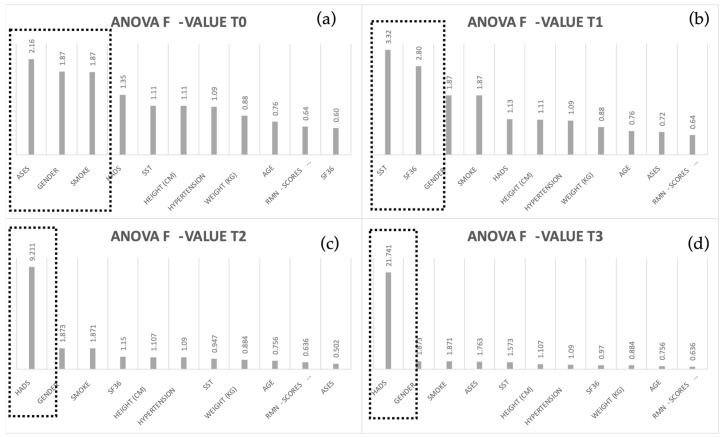
Features selected by performing statistical FS. (**a**) ASES, gender, and smoking were selected in T0, (**b**) SST and SF-36 were selected in T1, (**c**) HADS was selected in T2, and (**d**) HADS was selected in T3.

The optimal subset of features was obtained by defining a breakpoint as the highest difference between consecutive scores and selecting only the predictors with a score greater than it. Figure 2 displays the scores obtained for each session and the features selected by implementing the previous procedure.

Before performing feature selection, a correlation matrix was extracted to quantify the association between pairs of variables. OOS, SPADI, and SST features were highly correlated (rho > 0.8) during each session, which is not surprising since they all provide insight about the level of shoulder pain perceived by the patient. In order to prevent multicollinearity problems, we decided to keep only the variable that correlated the most with the output, which was the SST scale.

## 3. Results

**Figure 3:**
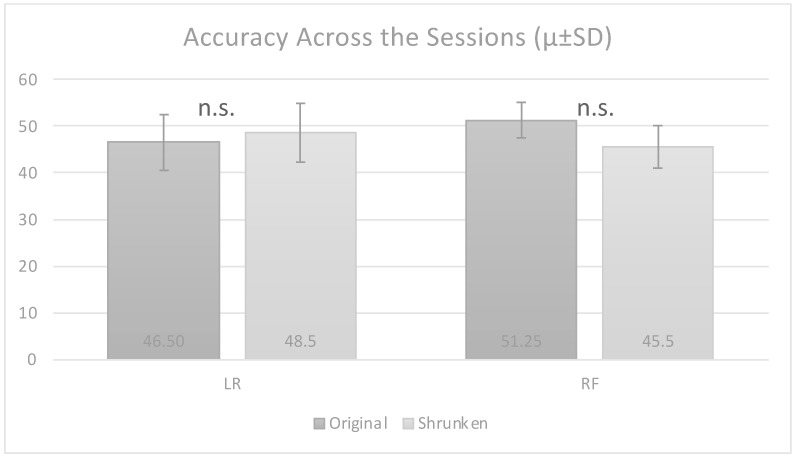
Visual comparison of accuracy scores achieved across sessions for both the original and shrunken datasets (µ ± SD).

No good performances were obtained for any of the classifiers during any of the sessions by using all the features, as detailed in Table 5. An accuracy greater than 50% was not achieved by any classifier across the sessions (Figure 3), resulting in a bad predictive capability of the ML models.

Following experimentation with various machine learning models it was observed that their performance remained comparable to that of the simplest logistic regression model. Consequently, we decided to present results solely for the logistic regression and random forest classifiers, the latter of which achieved superior overall accuracy.

We found the same results after performing feature selection, obtaining an accuracy of less than 55% for all sessions (Table 6). Notably, no increasing performances were obtained by using the shrunken set of features, as displayed in Figure 3.

Considering the obtained results, we conducted a Mann–Whitney U test to directly compare the accuracy of the two classifiers, namely, logistic regression and random forest. No statistically significant differences in the performance were found between the two models, as shown in Figure 3. Despite extensive exploration of various classifiers and feature sets, the models struggled to surpass a certain accuracy threshold, highlighting the challenges faced in achieving satisfactory predictive capabilities.

## 4. Discussion

Artificial intelligence (AI) has the ability to completely transform the practice of medicine by increasing precision and effectiveness, cutting costs, and delivering individualized treatment regimens [53]. In the orthopedic field, AI is being used to develop clinical prediction models for orthopedic patients that can help doctors make more informed decisions about patient care. Algorithms have been used to predict the outcomes of total joint replacement surgery [54], to predict fracture risk in orthopedic patients [55], and to diagnose orthopedic conditions such as osteoarthritis and spinal stenosis. To reach this performance level, AI algorithms need to analyze multidimensional patient data, such as age, medical history, motion, pain levels, medication therapy, bone density, and medical images such as X-rays and MRI scans, in order to recognize patients at higher risk for complications or poor outcomes after surgery.

In this study, we demonstrated that clinical data alone are not useful to reach a robust outcome prediction. Indeed, by using demographic information, comorbidities, and clinical metric scores, we were able to train a classifier with 55% accuracy at most in the third session.

According to the literature, another important function of ML approaches in the clinical setting appears to be the diagnostic one. This is confirmed by Ho et al. [56], who evaluated 194 ultrasound images of RCTs by fine-tuning pre-trained deep models in order to analyze and classify the RCTs. Among them, DenseNet121 demonstrated the best classification performance, with 88.2% accuracy, 93.8% sensitivity, 83.6% specificity, and an AUC score of 0.832. Kim et al. [57] developed a deep learning algorithm to rule out significant RCT based on conventional shoulder radiographs in 1095 shoulders suspected of RCT. The sensitivity of this approach was 97.3%, and it could rule out significant RCT with a negative likelihood ratio of 0.06 and a negative predictive value of 96.6%.

Concerning the reliability of clinical data as outcome measures of orthopedic surgical interventions, recently, Martin et al. [58] combined datasets from the Norwegian and Danish knee ligament registers in order to create an algorithm that can predict outcomes after primary anterior cruciate ligament reconstruction (ACLR). Using clinical data from 63,000 patients, ML analysis enabled prediction of the ACLR risk with moderate accuracy (68%). However, other publications found in the current literature positively identified significant clinical predictors. In fact, the research conducted by Li et al. [59] reported that ML models, in particular the XGBoost model, successfully recognized important clinical variables for the prediction of outcomes of 417 patients with RCTs. The most statistically significant values for prediction were the Jobe test, Bear hug test, and the age of the patients, with mean Shapley additive explanation (SHAP) values of 1.458, 0.950, and 0.790, respectively. Similarly, Dong et al. [60] studied a cohort of 1967 patients through a human–computer interactive Electronic Medical System (EMS) and demonstrated the presence of predictors of RC calcific tendinitis stratified according to the patients’ sex: women diagnosed with diabetes mellitus and men diagnosed with hyperlipidemia, diabetes mellitus, and hypothyroidism showed a higher risk of developing RC calcific tendinitis. Clinical factors such as age and sex were investigated in both articles, similarly to this paper. However, in the present paper, these features did not represent significant outcome predictors.

On the other hand, the most common functional outcomes reviewed as clinical outcome predictors by other articles in the current literatures were ASES, Visual Analog Scale (VAS), University of California Los Angeles (UCLA) Shoulder Score, CMS, and ROM. This is illustrated by Potty et al. [61] and Kumar et al. [62]. The former [61] applied the XGBoost algorithm to 631 patients undergoing arthroscopic RC repair to produce an expected post-operative ASES score. The anticipated improvement in the ASES score was within the Minimal Clinical Important Difference (MCID) value, indicating that ASES, even though the predictions did not exactly correspond to the actual results, along with BMI, age, and tendon quality, is a key predictor of clinical outcomes [9]. The latter [62], aiming to quantify the accuracy of prediction of postoperative ASES, UCLA, CMS, VAS, and ROM on 6210 patients, employed 3 different ML approaches: linear regression, XGBoost, and Wide and Deep. The Wide and Deep technique was associated with the smallest mean absolute error and predicted the postoperative ASES score to ±10.1 to 11.3 points, the UCLA score to ±2.5 to 3.4, the CMS to ±7.3 to 7.9, and the VAS pain score to ±1.2 to 1.4, demonstrating that preoperatively recognizing which patient characteristics may be predictive of a worse clinical outcome and issues of major clinical benefit improvement allows ML approaches to accurately risk-stratify patients. Moreover, the study by Vassalou et al. [63] demonstrated the validity of XGboost, which achieved an AUC of 69.2% (95%CI from 54.5% to 83.8%) for the prediction of complete resolution of pain at 1 year for 100 patients undergoing ultrasound-guided percutaneous irrigation of calcific tendinopathy of RC. Age, VAS, and the size of the calcification were found to be the three most important variables for the classification performance.

The present study evaluated various clinical scales, such as the SST, HADS, SF-36, OSS, and SPADI, which were not found in other publications on the same topic. However, it also assessed the ASES score, which was common to Potty et al. [61] and Kumar et al.’s publications [62]. Nonetheless, differently from the other articles, this paper did not find important predictive values of the ASES score, nor of the other clinical scales and clinical factors proposed.

The potential application of a predictive model based on subjective clinical scales such as the SST, HADS, SF-36, OSS, SPADI, and ASES would allow a deeper understanding of the patients’ current own health perception and allow the clinicians to better organize the treatment plan.

The lack of a significant application of AI algorithms to clinical prediction of RC patients could be dependent on several factors. The main limitation concerning the present paper is the small number of the cohort. However, it is important to note that research with similar inclusion and exclusion criteria is scarce within the existing literature. On the other hand, the data quality within the cohort poses additional limitations. Certain variables, such as smoking, diabetes, and work injury, are known to have an impact on the outcome. However, due to the nature of data collection, it was not feasible to include all relevant variables in the database. Moreover, some variables within the dataset have inherent uncertainties. For instance, the Goutallier grade, used to assess a specific aspect of the data, exhibits poor interrater reliability. The lack of consensus among raters regarding the Goutallier grade introduces ambiguity and inconsistency, which can adversely affect the performance of algorithms employed for predictions. In addition, we did not record other relevant clinical indicators such as diagnosis, implant type, ROM, and radiographic findings [64]. Another limitation is due to the fact that the three classes were not well balanced (Figure 1), which can cause the estimator to incorrectly discern the classes. Finally, the type of predictors, such as continuous and categorized (operator-dependent) variables, and the lack of objective biological high-dimensional data (i.e., neuroimaging, genetics), might have also limited the performance of our ML approach with respect to other studies.

## 5. Conclusions

We found that ML algorithms are not able to predict outcomes of patients with RC with sufficient accuracy using only clinical data. In fact, utilizing demographic data, comorbidities, and well-known clinical metrics, we were able to train a classifier session with a maximum accuracy of 55%. Despite that AI application in orthopedic surgery is in its relative infancy, our negative findings highlight the need to consider metrics capturing dynamic changes in prognosis, extending the current models with new objective predictors, such as kinematic and neuroimaging data. Computer-aided diagnostics may improve doctors’ ability to correctly identify musculoskeletal disorders and enhance the patients’ overall experience.

Finally, this study lays solid foundations for future studies to be carried out with greater cohorts and additional clinical scales.

## Figures and Tables

**Table 1:** Demographic information and clinical characteristics of the patients.

Predictors	RC (n = 102)
Age (years)	60.21 ± 7.77
Sex	40 F/60 M
Height (cm)	169.61 ± 8.09
Weight (kg)	76.38 ± 10.93
Smoking	20 Yes/80 No
Arterial Hypertension	52 Yes/48 No
Goutallier Grade of Rotator Cuff Muscle Degeneration (%)	
Stage 0	0%
Stage 1	72%
Stage 2	27%
Stage 3	0%
Stage 4	1%

**Table 2:** Conversion between Constant-Murley Score and corresponding qualitative classes.

Constant–Murley Score Conversion	
Range	Class
86–100	Very Good
71–85	Good
56–70	Fair
<56	Poor

**Table 3:** Hospital Anxiety and Depression Scale (HADS) Scoring and Classification.

Score	Anxious and Depressive State
8–10	Mild
11–14	Moderate
15–21	Severe

**Table 4:** Clinical scales of the patients at admission and during follow-up sessions.

Variable	µ(SD) T0	µ(SD) T1	µ(SD) T2	µ(SD) T3	Friedman Test (*p*-Value)
SF-36	100.34 ± 6.92	97.87 ± 7.59	103.24 ± 6.61	103.61 ± 6.12	<0.001
SST	3.80 ± 2.74	1.95 ± 2.22	6.82 ± 2.59	8.18 ± 2.86	<0.001
HADS	10.06 ± 7.47	7.76 ± 6.32	7.61 ± 7.68	8.59 ± 8.34	<0.001
ASES	36.34 ± 17.14	37.72 ± 13.62	64.33 ± 16.34	71.95 ± 10.26	<0.001
OSS	33.85 ± 10.39	39.87 ± 9.44	23.10 ± 8.06	18.40 ± 5.75	<0.001
SPADI	78.15 ± 26.41	88.75 ± 26.47	41.80 ± 23.39	31.88 ± 18.57	<0.01

**Table 5:** Overview of the performance metrics obtained from the application of the logistic regression (LR) and random forest (RF) classifiers using Nested CV across multiple sessions (T0–T3). Results are reported as percentage mean, along with the corresponding standard deviation, of the classifiers’ performance.

	Full Dataset		Classification Model	
Session	Metric (%)	Severity	LR	RF
T0	Accuracy	Overall	39 ± 12	46 ± 7
	Precision	Overall	46 ± 23	59 ± 13
	Recall	Overall	39 ± 12	46 ± 7
		Poor	5 ± 15	0 ± 0
		Fair	61 ± 28	77 ± 16
		Good	37 ± 15	38 ± 19
	F1-Score	Overall	34 ± 11	39 ± 6
	AUC	Overall	53 ± 16	57 ± 13
T1	Accuracy	Overall	49 ± 13	51 ± 10
	Precision	Overall	58 ± 16	61 ± 19
	Recall	Overall	49 ± 13	51 ± 10
		Poor	5 ± 15	0 ± 0
		Fair	62 ± 22	75 ± 23
		Good	62 ± 23	57 ± 25
	F1-Score	Overall	44 ± 12	44 ± 9
	AUC	Overall	62 ± 10	67 ± 8
T2	Accuracy	Overall	45 ± 15	54 ± 14
	Precision	Overall	47 ± 18	59 ± 16
	Recall	Overall	45 ± 15	54 ± 14
		Poor	38 ± 21	48 ± 24
		Fair	52 ± 27	55 ± 30
		Good	43 ± 22	58 ± 26
	F1-Score	Overall	44 ± 15	52 ± 15
	AUC	Overall	60 ± 16	69 ± 7
T3	Accuracy	Overall	53 ± 13	54 ± 14
	Precision	Overall	60 ± 15	57 ± 17
	Recall	Overall	53 ± 13	54 ± 14
		Poor	53 ± 28	45 ± 36
		Fair	57 ± 24	52 ± 27
		Good	51 ± 19	65 ± 23
	F1-Score	Overall	52 ± 12	51 ± 17
	AUC	Overall	69 ± 10	72 ± 12

**Table 6:** Overview of the performance metrics obtained from the application of the logistic regression (LR) and random forest (RF) classifiers using Nested CV across multiple sessions (T0–T3) after feature selection. Results are reported as percentage accuracy, along with the corresponding standard deviation, of the classifiers’ performance.

	Shrunken Dataset		Classification Model	
Session	Metric (%)	Severity	LR	RF
T0	Accuracy	Overall	48 ± 7	45 ± 7
	Precision	Overall	62 ± 15	56 ± 13
	Recall	Overall	48 ± 7	45 ± 7
		Poor	5 ± 15	5 ± 15
		Fair	82 ± 15	71 ± 27
		Good	36 ± 12	41 ± 12
	F1-Score	Overall	42 ± 7	39 ± 5
	AUC	Overall	60 ± 13	61 ± 13
T1	Accuracy	Overall	51 ± 8	46 ± 12
	Precision	Overall	66 ± 13	55 ± 19
	Recall	Overall	51 ± 8	46 ± 12
		Poor	0 ± 0	5 ± 15
		Fair	71 ± 18	56 ± 24
		Good	59 ± 29	62 ± 25
	F1-Score	Overall	43 ± 9	40 ± 11
	AUC	Overall	63 ± 13	59 ± 13
T2	Accuracy	Overall	40 ± 10	40 ± 14
	Precision	Overall	56 ± 14	43 ± 19
	Recall	Overall	40 ± 10	40 ± 14
		Poor	17 ± 21	48 ± 28
		Fair	62 ± 29	39 ± 32
		Good	32 ± 33	39 ± 24
	F1-Score	Overall	33 ± 13	37 ± 16
	AUC	Overall	62 ± 15	59 ± 11
T3	Accuracy	Overall	55 ± 11	51 ± 14
	Precision	Overall	61 ± 11	57 ± 13
	Recall	Overall	55 ± 11	51 ± 14
		Poor	33 ± 32	38 ± 37
		Fair	58 ± 18	46 ± 25
		Good	71 ± 22	67 ± 25
	F1-Score	Overall	53 ± 13	57 ± 17
	AUC	Overall	76 ± 9	74 ± 10

## Data Availability

The datasets used and/or analyzed during the current study are available from the corresponding author upon reasonable request. Access to the database is available upon request.
